# Separating ECog scale-score information from dyad availability: observation-process contributions to dyadic prediction of cognitive impairment in ADNI

**DOI:** 10.21203/rs.3.rs-10884877/v3

**Published:** 2026-09-22

**Authors:** Wan Kiu Winkey Cheng, for the Alzheimer’s Disease Neuroimaging Initiative

**Affiliations:** 1 Department of Biomedical Engineering, College of Biomedicine, City University of Hong Kong, Kowloon Tong, Hong Kong SAR, China

**Keywords:** subjective cognitive decline, dyadic informant report, ECog, prognostic model

## Abstract

**Background:**

Self- and informant-reported cognitive concerns are common, but their apparent incremental discriminative contribution can be inflated when missingness is structured by study protocol and assessment era. We tested the hypothesis that dyadic ECog would improve discrimination beyond conventional predictors, but that this increment would be attenuated after accounting for dyad availability and protocol-structured missingness.

**Methods:**

We included 1,643 cognitively unimpaired (CU) participants from the Alzheimer's Disease Neuroimaging Initiative (ADNI; 221 events: 213 mild cognitive impairment [MCI], 8 Alzheimer's disease [AD] dementia) and fit nested cross-validated elastic-net Cox models with fold-wise imputation.

**Results:**

The conventional model (M2) concordance index (C-index) was 0.680, but fell to 0.649 when missingness indicators were removed. M3 (M2 plus dyadic Everyday Cognition (ECog) mean and dyad availability) reached 0.758; after accounting for dyad availability, ECog scale scores showed a modest contribution beyond availability (ΔC = 0.026, 95% confidence interval (CI) 0.007 to 0.045), whereas the score-only comparison provided limited evidence for an independent contribution (ΔC = 0.010, 95% CI −0.004 to 0.024). In an inverse-probability-of-censoring-weighted (IPCW) sensitivity analysis, ΔC was 0.105 (95% CI 0.050 to 0.174).

**Conclusions:**

In this ADNI analysis, missingness-indicator imputation may increase the apparent discriminative contribution of dyadic measures; much of the observed gain was associated with dyad availability, and limited evidence was found for an independent contribution from ECog scale scores.

## Introduction

Cognitively unimpaired older adults at risk of incident cognitive impairment are an important target for prognostic modelling and trial enrichment. Self- and informant-reported concerns are common, and discordance is associated with MCI diagnosis [1]. They are also associated with severity and progression [2-9], but this does not establish incremental discrimination beyond an information-rich model. We therefore ask whether a single baseline dyadic summary improves discrimination once conventional demographic, cognitive, genetic, amyloid, and neurodegeneration predictors are already available. A dyadic summary may reflect perceived difficulty, whereas discordance may reflect awareness or appraisal [1,3-5]. In this study, we operationalised these constructs as the dyadic ECog mean and a linear self–informant gap. Their contribution may depend on disease stage and how report availability is determined. Availability may therefore reflect assessment-era structure as well as progression.

The Everyday Cognition (ECog) scale is a structured self- and informant-report instrument that samples day-to-day cognitive difficulties across multiple domains [10-11]. We used ADNI-supplied 39-item ECog summary fields, scored as the mean of completed items; the later 12-item short form does not populate these fields, so no cross-form mixing occurred [10-11]. Subjective cognitive decline is a research construct associated with cognitive decline [12-13] and later clinical progression and severity [3-4,6-8]. Conventional predictors such as APOE4 carrier status, amyloid-PET Centiloid values, and hippocampal volume are established Alzheimer-related risk, pathological-staging, and neurodegeneration markers [14-18]. Whether any observed gain from adding a baseline dyadic ECog summary reflects ECog scale-score information or the observation process that determines dyad availability, however, remains unclear [5,8].

We conducted an exploratory study with prespecified analyses in ADNI. The principal objective was to distinguish discriminative information carried by ECog scale scores from information carried by dyadic-report availability under the ADNI assessment structure; the secondary objective was to quantify the ECog increment for incident MCI or AD dementia beyond observation-process structure. The model hierarchy (M0, M2, M3, M4; M1 was reserved for a non-biomarker model and not fitted), primary contrast (M3 versus M2), and fold-wise workflow were specified before the locked run. A post-hoc sensitivity additionally refitted M2 without missingness indicators. Reporting follows TRIPOD standards.

## Methods

### Study design and participants

We included 1,643 ADNI participants who were cognitively unimpaired (CU) at the landmark visit (typically the screening visit; Supplemental Figure 1). The landmark was the earliest ADNI visit at which a participant was classified as CU; baseline covariates were drawn from that visit, and biomarkers used the closest measurement within ±180 days. Participants with no post-landmark visit were censored at t=0. Eligibility required a baseline CU diagnosis; when none was recorded, CDR=0 served as fallback; a recorded non-normal diagnosis was exclusionary. A global CDR of 0 and MMSE ≥ 24 were applied only when recorded. Landmark-year bands served as an assessment-era proxy. The primary endpoint was the first clinical diagnosis of MCI or AD dementia after the landmark; events were 213 MCI and 8 AD dementia, and 1,422 participants were censored, of whom 373 (22.7% of the full cohort) had no post-landmark visit. Death was treated as right-censoring; competing-risk models were not fitted. We used the first recorded diagnosis date; interval-censored placements were not used [19]. ADNI is a longitudinal multi-site study launched in 2003 [20-22]; files were downloaded on 31 January 2026, with follow-up through 3 November 2025.

Data used in the preparation of this article were obtained from the ADNI database (adni.loni.usc.edu), a public-private partnership launched in 2003 and led by Principal Investigator Michael W. Weiner, MD (www.adni-info.org).

### Predictors

Model M0 (clinical-demographic baseline) included age, sex, education, and MMSE. Each model included missingness indicators for predictors subject to missingness (age, education, MMSE, APOE4 carrier status, Centiloid, and hippocampal volume); female sex (one missing value) was median-imputed without a separate indicator. Model M2 (conventional-predictor model) extended M0 with APOE4 carrier status, amyloid-PET Centiloid values, and hippocampal volume. Post-hoc M2 without missingness indicators tested observation-process structure. Hippocampal volume was the ADNI-derived bilateral total in mm^3^ without intracranial-volume adjustment. Biomarkers were anchored to the landmark by selecting the closest measurement within ±180 days; for participants who later experienced the primary event, only pre-event measurements were eligible. The ±180-day window is a retrospective research convenience, so temporal relationships are approximate. The full predictor list and model hierarchy are documented in Supplemental Table 8.

ECog self- and informant-report total scores were the ADNI-derived, precomputed mean of completed 39-item form items (1-4 Likert; higher totals indicate greater concern) [10-11]. The dyadic ECog mean and gap were calculated only when both scores were available; otherwise they were median-imputed within each outer-training fold and accompanied by the dyad-availability indicator, with no separate score-missingness indicators. The linear self–informant discrepancy (gap = informant minus self) was prespecified as the primary discrepancy measure; non-linear or categorical profiles were not included and are discussed as future directions.

Baseline-predictor missingness was associated with assessment era, protocol evolution, and scan availability. We used fold-wise median imputation within each outer-training fold, projected to test data, with missingness indicators for age, education, MMSE, APOE4 carrier status, Centiloid, and hippocampal volume; the dyadic ECog mean and gap used the dyad-availability indicator. Multiple imputation was avoided because the objective was discrimination under the observed ADNI process, not etiological estimation [23-25]. The missing-indicator approach was prespecified but can introduce bias when missingness is protocol-structured [25]; coefficients should be read as empirical adjustment weights [23-25]. A complete-dyad analysis (both reports observed; n=418, events=54) provided an imputation-free conditional benchmark under the same fold-wise procedures.

### Statistical analysis

We fit elastic-net Cox survival models with L1 mixing parameter (α = 0.5) [26-29]; the penalty λ was selected from {0.001, 0.005, 0.01, 0.05, 0.1} by inner 3-fold cross-validation. All preprocessing was fit inside each outer-training fold only. Discrimination used Harrell’s concordance index on pooled out-of-fold (OOF) linear predictions [30-31]. Under Harrell’s C-index, participants censored at time 0 (n=373) contribute uninformative ties; because dyad availability was associated with follow-up length, the availability indicator may inflate Harrell’s C-index by separating long-follow-up from time-0-censored participants. We therefore additionally computed Uno’s IPCW concordance index [32], which reweights pairs by estimated censoring probability and is less sensitive to the marginal follow-up distribution; the censoring distribution was estimated once on the analysis sample and held fixed across bootstrap resamples. This reduces but does not eliminate concern about unequal censoring. Decision-curve analysis was not performed because the model was not an absolute-risk calculator.

The event-stratified outer 5-fold loop provided a reduced-optimism internal estimate of discrimination, while the inner 3-fold loop selected the penalty parameter without touching the outer test fold [33]. Preprocessing was fit on outer-training folds and applied to test folds; because development and evaluation used one ADNI cohort, performance may be optimistic [34-35]. Survival analyses used the scikit-survival Python package.

Paired bootstrap contrasts (1,000 replicates) compared OOF C-index values between models. Bootstrap confidence intervals were calculated by resampling participants' paired out-of-fold predictions, taking the 2.5th and 97.5th percentiles of the bootstrap distribution, and therefore quantify uncertainty conditional on the fitted cross-validation pipeline; they do not fully capture model-selection uncertainty from the inner cross-validation loop.

### Proportional-hazards and sensitivity diagnostics

We evaluated proportional hazards with Schoenfeld residual tests on rank-transformed event times for M3 and M4 [36], using a ridge-stabilized Cox fit (penalty 0.05) over the full predictor set (Supplemental Table 7; Supplemental Figure 5). Hippocampal volume showed borderline non-proportionality (p = 0.046), so Cox models are used to compare relative discrimination rather than to provide stable hazard ratios. Sensitivity analyses comprised: M2 without missingness indicators; an availability-only decomposition; a score-only contrast (M3 without the dyad-availability indicator versus M2); IPCW C-index comparison; interval-censoring analysis; a complete-dyad subset; a minimum follow-up restriction (≥0.5 years); and a protocol-era model with landmark-year bands. A repeated nested-CV split-stability analysis is summarized in Supplemental Table 6 and Supplemental Figure 4.

## Results

### Cohort characteristics and missingness

Median time to event or censoring was 1.24 years (IQR 0.05–5.86; range 0.00–19.23); median event time 3.62 years (IQR 1.54–6.48); median censoring time 0.81 years (IQR 0.00–5.46); total person-years 5,134.7. Early censoring was substantial, reflecting the ADNI visit schedule and limited follow-up among cognitively unimpaired participants (Table 1; Supplemental Tables 1 and 2; Supplemental Figure 2).

Dyadic ECog data were available for approximately 25% of the cohort, reflecting structural unavailability of ECog scale scores under phase-specific administration rather than uniform participant nonresponse. APOE4 carrier status was available for 69% of participants; amyloid and hippocampal imaging were available for approximately 55% and 52%, respectively, after anchoring measurements within a ±180-day window of the landmark date.

### Primary model discrimination

The primary contrast (M3 versus M2) showed an increment in internally cross-validated discrimination whose bootstrap CI excluded zero (ΔC = 0.078, 95% CI 0.050 to 0.106; M2 0.680, M3 0.758; Table 2, Figure 1). A post-hoc sensitivity analysis showed that the conventional-predictor model's discrimination decreased to 0.649 when missingness indicators were omitted (Table 3). M3 combines the observed dyadic ECog mean with a dyad-availability indicator that captures whether a complete dyad was available under the historical ADNI protocol. This full feature-set result should be interpreted as an operational association within the observed ADNI measurement process rather than as an independent clinical effect. Adding the self–informant gap (M4 versus M3) did not improve discrimination beyond the dyadic ECog mean model (ΔC = −0.004, 95% CI −0.008 to 0.001).

### Sensitivity analyses of incremental discrimination

Dyadic data were available for about 25% of participants (Supplemental Table 1). M2 without missingness indicators had C-index 0.649, 0.032 lower than conventional M2 (0.680; Table 3). Adding only the dyad-availability indicator raised the C-index from 0.680 to 0.733 (ΔC = 0.052, 95% CI 0.023 to 0.082). After accounting for availability, ECog scale scores showed a modest contribution (ΔC = 0.026, 95% CI 0.007 to 0.045), whereas the score-only comparison provided limited evidence for an independent contribution (ΔC = 0.010, 95% CI −0.004 to 0.024). Interval-censoring and sensitivity analyses were concordant (Supplemental Tables 3–4). Coefficients are in Supplemental Table 9. Uno’s IPCW C-index yielded M2 0.687 and M3 0.792 (ΔC = 0.105, 95% CI 0.050 to 0.174); this reduces but does not remove protocol-driven censoring, because availability is also associated with protocol era, visit completion, biomarker acquisition, and longer follow-up. In the complete-dyad subset (n=418, events=54) the increment was larger (ΔC = 0.190, 95% CI 0.091 to 0.293), but M0 (0.452) and M2 (0.535) were attenuated, partly reflecting poor baseline discrimination rather than a reliable isolated ECog effect. Descriptive analyses suggested potential risk stratification in this selected sample (Supplemental Section S3; Supplemental Figure 3). Minimum-follow-up and protocol-era analyses were consistent. Split-stability and PH diagnostics are in Supplemental Tables 6 and 7 and Supplemental Figures 4 and 5; the MRI availability analysis is in Supplemental Table 5.

## Discussion

In ADNI, the dyadic ECog feature set raised the out-of-fold C-index to 0.758, but this should not be interpreted as a clinically applicable tool. Without missingness indicators, M2 fell from 0.680 to 0.649. Much of the 0.078 increment was associated with dyad availability; after accounting for availability, ECog scale scores showed a modest increment (ΔC = 0.026, 95% CI 0.007 to 0.045), whereas the score-only estimate was ΔC = 0.010 (95% CI −0.004 to 0.024). The former separates score from availability–follow-up, whereas the latter conflates them. Score evidence depended on availability representation, indicating entanglement. The self–informant gap did not improve discrimination. Self- and informant-reported concerns may remain informative, but registry missingness may inflate their apparent contribution.

Dyadic reports may differ across cognitive stages and discrepancy direction [1,6-8], and have been associated with Alzheimer’s biomarkers across preclinical and prodromal states [37] and with longitudinal cognitive outcomes [38]. Self–informant discrepancy may be bidirectional: heightened self-concern may occur early, whereas informant-predominant concern may emerge near clinical transition [5,8,12]. A linear gap may fail to capture these dynamics; categorical or profile-based features may be more appropriate. Preserved awareness may precede reduced insight (anosognosia), shifting concern toward informants as neurodegeneration progresses [12-13]. In ADNI, the static summary also carried availability information; the null score-only result should not be read as evidence that ECog lacks discriminative contribution.

The limited incremental contribution of the linear dyadic summary does not imply limited clinical relevance of subjective cognitive complaints [12-13]. Self- and informant-reported concerns may reflect partly distinct neurocognitive constructs that evolve across the Alzheimer’s continuum: preserved insight in preclinical stages, reduced self-awareness with advancing neurodegeneration, and informant-predominant concern in later stages [5,8,12]. ECog was designed to capture subtle everyday difficulties that may precede measurable decline [10-11]. The dyadic mean showed a modest contribution (ΔC = 0.026), while the linear gap did not improve discrimination, suggesting that non-linear, stage-dependent awareness transitions may require categorical or profile-based features. These findings evaluate one prognostic representation, not dyadic assessment itself. Future studies should examine awareness transitions in cohorts with repeated dyadic measures and biomarker staging.

Dyadic ECog was available for about 25% of the cohort, and availability tracked assessment era, visit completion, biomarker acquisition, and follow-up: available dyads had higher biomarker availability (amyloid-PET 82.3% versus 45.1%; hippocampal volume 77.8% versus 43.3%; Supplemental Table 1) and longer follow-up (median 7.0 versus 0.26 years). Longer observation creates more opportunity for events, so availability is not a patient-level predictor. The availability-adjusted increment was 0.026; score-only evidence included the null.

The conventional model is conditioned on the observed ADNI process. The primary contrast was M2 0.680 versus M3 0.758 (ΔC = 0.078); availability alone reached 0.733, and score beyond availability added 0.026. The span from M2 without missingness indicators (0.649) to full M3 (0.758) was 0.110; this asymmetric contrast should not be confused with the Table 3 M2-without-missingness increment (0.032). Uno IPCW gave ΔC = 0.105, indicating that the association was not eliminated after IPCW adjustment; residual protocol-related selection and observation-process effects remain possible. The complete-dyad ΔC = 0.190 was unstable due to attenuated baseline discrimination. Future studies should compare universal administration and explicit missingness models.

A strength is the prespecified workflow with fold-wise preprocessing and fixed cross-validation folds. The pipeline uses fixed seeds and a source-keyed results registry. A completed TRIPOD checklist (Supplemental Table 10) and archived analysis code support independent verification and reproducibility [39-40].

The study has limitations. First, it is an internally cross-validated analysis requiring external validation [34-35]. Second, structural unavailability of dyadic reports reflects historical cohort design and cannot be fully eliminated by missingness indicators or complete-dyad analyses [23,25]. Third, median time to event or censoring was 1.24 years and 373 participants (22.7% of the full cohort) had no post-landmark visit, producing uninformative ties under Harrell’s C-index. Fourth, only 39-item ECog scores were analysed; generalizability to the 12-item short form is not established. Fifth, diagnostic adjudication may overlap with informant-reported complaints, so incorporation bias cannot be excluded. Sixth, death was treated as right-censoring; cause-specific or Fine–Gray competing-risk models could be considered depending on the estimand of interest. Seventh, landmark-year bands are administrative proxies; persistence of the increment after their inclusion is compatible with residual temporally patterned assessment effects, but does not distinguish these from genuine score information. Eighth, hippocampal volume showed borderline non-proportionality (p = 0.046). Ninth, dyadic ECog was available for only 25% of participants. Tenth, missingness mechanisms were not modelled, so score and observation-process contributions cannot be estimated causally. Finally, sensitivity contrasts were not adjusted for multiplicity, calibration was not evaluated, and IPCW intervals do not reflect censoring-distribution uncertainty. These models do not provide clinical risk estimates.

These findings caution prognostic studies using registry data: the reported performance reflects prediction under the ADNI observation process, and the evidence was modest and model-dependent. Future studies should distinguish ECog scale-score information from observation-process effects through universal administration or explicit missingness models. The availability-only comparison was the largest increment, the availability-adjusted score increment was small (95% CI 0.007 to 0.045), and the score-only estimate included the null. These findings should not be interpreted as proof that ECog cannot make a discriminative contribution under alternative designs; its contribution under properly modelled missingness remains an open question for external validation.

## Supplementary Material

This is a list of supplementary files associated with this preprint. Click to download.

- supplemental1.docx
- supplementalfigure5.png
- supplementalfigure1titled.png
- supplementalfigure4.png
- supplementalfigure2.png
- supplementalfigure3.png

## Figures and Tables

**Figure 1.**
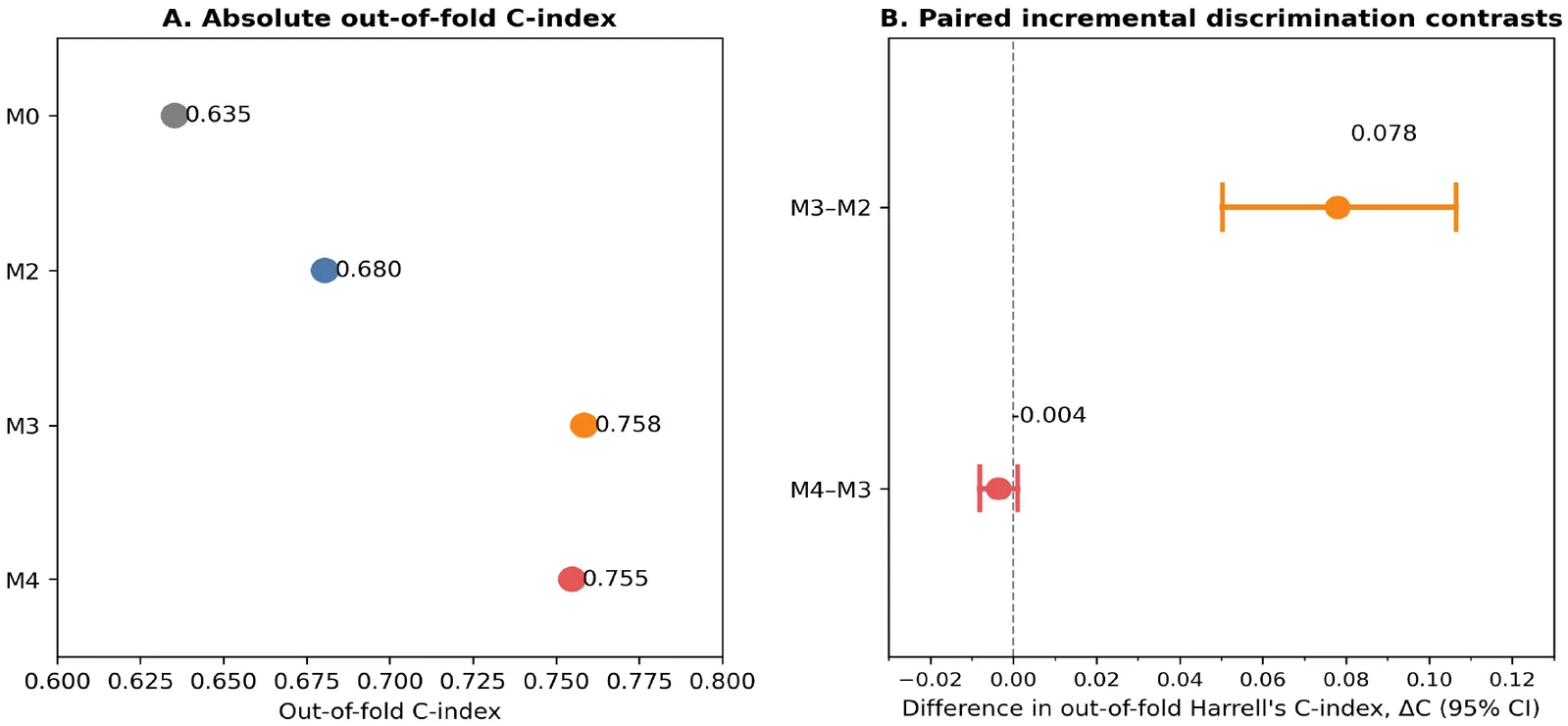
Out-of-fold discrimination across prespecified models and paired incremental discrimination contrasts. A, pooled out-of-fold Harrell's C-index for the clinical-demographic baseline (M0), conventional-predictor model (M2), M2 plus mean dyadic ECog scale scores and the dyad-availability indicator (M3), and M3 plus the self–informant gap (M4). B, paired bootstrap contrasts (M3–M2, M4–M3) with percentile 95% confidence intervals from 1,000 replicates; the dashed line denotes no difference. The M3–M2 contrast reflects the full feature-set model (dyadic ECog scale scores plus the observable availability of a complete dyad); adding the gap did not improve discrimination.

**Table 1.** ADNI cohort characteristics and predictor missingness. AD, Alzheimer disease; APOE, apolipoprotein E; ECog, Everyday Cognition; MCI, mild cognitive impairment; MMSE, Mini-Mental State Examination; SD, standard deviation. Continuous variables: mean (SD); categorical: n (%). Percent missing is relative to the full n=1,643 cohort; high missingness for ECog, amyloid, and MRI reflects multi-phase ADNI protocols rather than uniform attrition. Binary amyloid positivity is shown for descriptive context and was not used as a model predictor.

Characteristic	N	Summary	% missing
Age, years	1642	70.69 (7.33)	0.06
Education, years	1640	16.49 (2.52)	0.18
MMSE	1639	29.01 (1.17)	0.24
Female, n (%)	1642	991 (60.4)	0.06
APOE4 carrier, n (%)	1133	369 (32.6)	31.04
Centiloid	897	17.04 (31.43)	45.40
Amyloid positive, n (%)	897	220 (24.5)	45.40
Hippocampal volume	856	8027.45 (847.84)	47.90
ECog self total	425	1.40 (0.37)	74.13
ECog informant total	420	1.18 (0.25)	74.44
ECog dyadic mean	418	1.29 (0.25)	74.56
ECog dyadic gap	418	−0.22 (0.39)	74.56

**Table 2.** Out-of-fold C-index for the locked ADNI models.

Model	N	Events	C-index
M0	1643	221	0.635
M2	1643	221	0.680
M3	1643	221	0.758
M4	1643	221	0.755

**Table 3.** Sensitivity analyses of incremental discrimination. The primary M3-M2 contrast was prespecified; the remaining analyses assess internal stability across follow-up, protocol era, and dyad availability. Rows statistically decompose the baseline effect of missingness indicators, the availability-only increment, the ECog scale-score increment beyond availability (M3 versus M2 plus the dyad-availability indicator), the score-only increment, and a censoring-robust IPCW contrast. For the minimum-follow-up row, participants with events before 0.5 years were retained to preserve the event count; N therefore comprises participants with at least 0.5 years of observed follow-up or an observed event before that threshold. Complete-dyad baseline models (M0 0.452, M2 0.535) are weak, so those estimates are unstable. ΔC values are computed on unrounded C-indices, so displayed differences may differ from displayed C-index values by ±0.001.

Sensitivity / analysis	N	Events	Reference C- index	Extended-model C-index	Extended–Ref ΔC (95% CI)
Primary (raw, full sample)	1643	221	0.680	0.758	0.078 (0.050 to 0.106)
M2 plus dyad-availability indicator	1643	221	0.680	0.733	0.052 (0.023 to 0.082)
ECog scale-score contribution beyond dyad availability (M3 vs M2 + dyad availability)	1643	221	0.733	0.758	0.026 (0.007 to 0.045)
M2 without missingness indicators	1643	221	0.649	0.680	0.032 (0.008 to 0.058)
Score-only model (M3 without dyad-availability indicator vs M2)	1643	221	0.680	0.690	0.010 (−0.004 to 0.024)
Uno's IPCW C-index (M3 vs M2)	1643	221	0.687	0.792	0.105 (0.050 to 0.174)
Complete-dyad subset	418	54	0.535	0.725	0.190 (0.091 to 0.293)
Minimum follow-up ≥0.5 years	971	221	0.681	0.761	0.080 (0.051 to 0.109)
Protocol-era (landmark-year band) candidate variables	1643	221	0.694	0.775	0.081 (0.052 to 0.113)
